# Loosely controlled experimental EEG datasets for higher-order cognitions in design and creativity tasks

**DOI:** 10.1016/j.dib.2023.109981

**Published:** 2023-12-18

**Authors:** Morteza Zangeneh Soroush, Mengting Zhao, Wenjun Jia, Yong Zeng

**Affiliations:** Concordia Institute for Information Systems Engineering, Gina Cody School of Engineering and Computer Science, Concordia University, Montreal, QC H3G 2W1, Canada

**Keywords:** Design creativity, Higher order cognitive tasks, Loosely controlled experiments, EEG, Torrance test of creative thinking (TTCT), NASA task load index (NASA-TLX), Creativity cognition, Design neurocognition

## Abstract

Understanding neural mechanisms in design and creativity processes remains a challenging endeavor. To address this gap, we present two electroencephalography (EEG) datasets recorded in design and creativity experiments. We have discussed the details, similarities, differences, and corresponding cognitive tasks of the two datasets in the following sections.

The design dataset (Dataset A) comprises EEG recordings of 27 participants during loosely controlled design creation experiments. Each experiment included six design problems. In each design problem, participants performed five cognitive tasks, including problem understanding, idea generation, rating idea generation, idea evaluation, and rating idea evaluation. The NASA Task Load Index was used in rating tasks.

The creativity dataset (Dataset B) includes EEG signals recorded from 28 participants in creativity experiments which were based on a modified variant of the Torrance Test of Creative Thinking (TTCT-F). Participants were presented with three incomplete sketches and were asked to perform three creativity tasks for each sketch: idea generation, idea evolution, and idea evaluation.

In both datasets, we structured the experiments into predefined steps, primarily to ensure participants' comfort and task clarity. This was the only control applied to the experiments. All the tasks were loosely controlled: open-ended (up to 3 min) and self-paced. 64-channel EEG signals were recorded at 500 Hz based on the international 10–10 system by the Brain Vision EEG recording system while the participants were performing their assigned tasks. EEG channels were pre-processed and finally referenced to the Cz channel to remove artifacts. EEGs were pre-processed using popular pipelines widely used in previous studies. Preprocessed EEG signals were finally segmented according to the tasks to facilitate future analyses. The EEG signals are stored in the .mat format. While the present paper mainly addresses pre-processed datasets, it also cites raw EEG recordings in the following sections. We aim to promote research and facilitate the development of experimental protocols and methodologies in design and creativity cognition by sharing these resources. There exist important points regarding the datasets which are worth mentioning. These datasets represent a novel contribution to the field, offering insights into design and creativity neurocognition. To our knowledge, publicly accessible datasets of this nature are scarce, and, to the best of our knowledge, our datasets are the first publicly available ones in design and creativity. Researchers can utilize these datasets directly or draw upon the considerations and technical insights provided to inform their studies. Furthermore, we introduce the concept of loosely controlled cognitive experiments in design and creativity cognition. These experiments strike a balance between flexibility and control, allowing participants to incubate creative ideas over extended response times while maintaining structured experimental sections. Such an approach fosters more natural data recording procedures and holds the potential to enhance the accuracy and reliability of future studies. The loosely controlled approach can be employed in future cognitive studies. This paper also conducts a comparative analysis of the two datasets, offering a holistic view of design and creativity tasks. By exploring various aspects of these cognitive processes, we provide an understanding for future researchers.

Specifications Table

**Table:** 

Subject	*Neuroscience (Cognitive)*
Specific subject area	*Cognitive Neuroscience in Design and Creativity Experiments* *Similar terms: Design Cognition, Creativity Neurocognition, Design Creativity, Design Neurocognition*
Data format	Two datasets including continuous pre-processed EEG signals (in .mat format) recorded during design and creativity tasks, respectively. These two datasets are called the design dataset (or dataset A) and the creativity dataset (or dataset B) in this paper
Type of data	*.mat files (pre-processed EEG signals recorded in design and creativity experiments)*
Data collection	*Participants signed an informed consent and received instructions. EEG data was recorded using a 64-channel BrainVision actiCHamp system (10-10 international system), sampled at 500 Hz, and referenced to the Cz which was later removed. Design and creativity experiments were loosely controlled, allowing self-paced, open-ended tasks (up to three minutes), leading to natural experiments. EEG signals were recorded while participants were performing the assigned design or creativity tasks. EEGs during two rest modes (3 min closed-eye periods at the beginning and end of experiments) were also recorded. Finally, recorded EEGs were pre-processed, segmented, and stored in the two datasets.*
Data source location	Design Lab Institution: Concordia University City: Montreal Province: Quebec Country: Canada Latitude and Longitude: 45.4948° N, 73.5779° W
Data accessibility	Design Dataset (Dataset A) Repository name: Mendeley Data Data identification number: DOI:10.17632/h4rf6wzjcr.1 Direct URL to data: https://data.mendeley.com/datasets/h4rf6wzjcr/1 Instructions for accessing these data: The title of this dataset is “Conceptual Design Exploration: EEG Dataset in Open-ended Loosely Controlled Design Experiments”. The data is open-access and EEG signals are stored in a folder named Design_EEG_Dataset. You can see 27 .mat files which contain EEG signals associated with 27 participants in the design experiments. All the EEG recordings, associated with that specific participant, are stored as variables in the corresponding .mat file. Creativity Dataset (Dataset B) Repository name: Mendeley Data Data identification number: DOI:10.17632/24yp3xp58b.1 Direct URL to data: https://data.mendeley.com/datasets/24yp3xp58b/1 Instructions for accessing these data: The title of this dataset is “Design Creativity: EEG Dataset in Loosely Controlled Modified TTCT-F Creativity Experiments”. The data is open-access and EEG signals are stored in a folder named Creativity_EEG_Dataset. You can see 28 .mat files which contain EEG signals associated with 28 participants in the creativity experiments. All the EEG recordings associated with that specific participant are stored as variables in the corresponding .mat file.
Related research article	Jia, W., von Wegner, F., Zhao, M. et al. Network oscillations imply the highest cognitive workload and lowest cognitive control during idea generation in open-ended creation tasks. Sci Rep 11, 24277 (2021). https://doi.org/10.1038/s41598-021-03577-1[1]

## 1 Value of the Data

• The datasets presented here comprise electroencephalographic (EEG) signals, offering the sole publicly accessible resource in the design and creativity neurocognition field. With 64 EEG channels, they provide high spatial resolution, a significant advantage over studies typically employing fewer channels. This high-density EEG data facilitates precise brain dynamics analysis in design and creativity tasks. The authors of the present paper explored brain dynamics in design and creativity tasks in [1], [2], [3], [4] using the present datasets. Their significant results approved the datasets and suggested that these datasets can effectively represent brain behavior and pave the way for future studies.
• Undoubtedly, high-quality EEG signals are required to investigate the brain's behavior. Collecting datasets is challenging as design and creativity processes are mainly open-ended and ill-structured. In addition, recorded EEG signals carry a considerable amount of noise and artifacts since design and creativity activities include physical movements. Denoising long EEG signals needs efficient methods due to their nonlinear and non-stationary behavior. We employed effective and reliable preprocessing methods based on popular and highly cited EEG preprocessing pipelines to achieve high-quality EEGs. So, this paper introduces two pre-processed and high-quality EEG datasets for design and creativity experiments.
• We provided segmented EEGs associated with the corresponding tasks in the datasets to facilitate future analyses. These datasets are valuable for researchers in neuroscience, design cognition, creativity, and machine learning. They include pre-processed and segmented EEG signals linked to various cognitive tasks in design and creativity experiments, such as problem understanding, idea generation, idea evolution, and idea evaluation, etc. The segmented and labeled EEGs help researchers easily explore brain dynamics in different cognitive tasks through EEGs. These datasets are well-suited for neuroscience and machine learning applications, allowing researchers to develop EEG processing methods or classify EEGs into cognitive states.
• Due to our limited knowledge of neural circuits in design and creativity, these datasets play a pivotal role in advancing the field of design cognition. They provide invaluable neural recordings, encouraging further exploration in this area. Researchers can use EEG signals from these datasets to correlate and cross-reference neuroscientific findings with established cognitive models and theories in design creativity. The scarcity of such datasets in these domains may explain the relative lack of research compared to other cognitive neuroscience fields. We introduce two datasets for design creativity in this paper, which recent studies like [1], [2], [3], [4] have validated.
• The two datasets were recorded using loosely controlled experiments, self-paced and open-ended with minimal control (the experiment steps), suited to the ill-defined nature of design and creativity tasks. This method provides more reliable results than strictly controlled experiments, which impose rigid timing on designers. Striking a balance between uncontrolled and strictly controlled settings, these experiments blend scientific controls with creative flow, allowing ample time for each step. Contrasting with traditional experiments that limit creativity and lack real-world relevance, loosely controlled experiments enhance EEG applications in design creativity studies, and enable a deeper understanding of interactions between design tasks.
• Design and creativity, while inseparable and closely intertwined cognitive tasks, present nuanced distinctions. This paper not only introduces and compares the datasets but also underscores their practical implications. It sheds light on designing protocols for complex, open-ended, and ill-structured cognitive experiments, particularly in the realms of design and creativity. The paper addresses cognitive processes underlying design creativity experiments, discusses the datasets' practical aspects, shedding light on considerations for EEG recording during design and creativity experiments. It provides a resource for researchers to access EEG recordings for both experiments, facilitating hypothesis testing related to the cognitive mechanisms of design and creativity.

## 2 Data Description

To prepare the readers for data descriptions, here we briefly explain design and creativity and the reason why we decided to present the two datasets in one article. Creativity has been reported to be inextricably linked to humanity's progress and even its survival. On a daily basis, people use furniture, services, and devices made by creative designers, suggesting the importance of a field called design creativity. Design creativity, which refers to designing creative products, has three standard criteria, including originality, effectiveness, and surprise [5]. The process of design is tightly intertwined with creativity. Designers digest problems and constraints and empathize with users to introduce effective and appropriate solutions. Creativity can be found in every single step of design and even as a criterion to judge designers’ work. This indicates that design and creativity are two inseparable processes [6,7]. This paper introduces two datasets in design creativity. The reason why two datasets, one focusing on design experiments and the other more on creativity tasks, are introduced in this paper mainly stems from the concept of design experiments and creativity, which are undeniably intertwined. In other words, design steps and the ones in creativity tasks have a lot in common, such as problem understanding, idea generation, evolution, and evaluation. We cannot equate design with creativity, as we do not expect designers to follow the same path in their design procedures. What makes a design creative is novelty, unexpectedness, and novel purposes [8]. Creativity, which is defined as developing novel solutions to problems, is similar to design, which is mainly defined as a discipline seeking a balance between originality and functionality, novelty and appropriateness. The way a designer strikes this balance locates creativity at the heart of a design process. In other words, creativity enables designers to innovate novel solutions from conventional knowledge domains by investigating new ideas and concepts [1,2,6,9]. The two datasets in this paper can pave the way for future studies focusing on design tasks or creativity experiments as two correlated higher-order cognitive tasks. In addition, researchers can compare the brain dynamics in design and creativity cognitive tasks through these two datasets. EEG signals in both datasets have been analyzed in some studies [1], [2], [3], [4] and have been reported to effectively describe the brain's dynamics in design and creativity tasks.

For simplicity, in this article, we name the design dataset and the creativity dataset as datasets A and B, respectively. In this section, we briefly mention datasets’ main characteristics, some general considerations, similarities, and differences in both datasets to provide a general overview. In addition to this general information about the datasets, we provide a description of the datasets in Table 1 for readers to consider differences and similarities simply. More detailed descriptions for both datasets are provided in the following sections.

**Table 1:** Description of design and creativity experiments, including general information, similarities, and differences.

	Design Experiment (Dataset A)	Creativity Experiment (Dataset B)
Participants	27 right-handed graduate students (8 women) aged from 24 to 39	28 right-handed graduate students (4 women) aged from 22 to 35
Recorded biosignals	500 Hz sampled 64-channel EEG signals based on the international 10–10 system and referenced to Cz, and later down-sampled to 250 Hz	64-channel EEG signals recorded at 500 Hz based on the international 10–10 system and referenced to Cz
Design problems	Six consecutive design problems including designing a birthday cake, a toothbrush, a workspace, a recycle bin, a wheelchair, and a drinking fountain	Three consecutive creativity problems (stimuli) derived from modified figural Torrance Test of Creative Thinking (TTCT-F) test including three incomplete sketches
Tasks included in each trial	problem understanding, idea generation, rating idea generation, idea evaluation, and rating idea evaluation	idea generation, idea evolution, and idea evaluation
Rating (Evaluation method)	NASA Task Load Index (five discrete rates from 0 to 100 with 5-point steps) including mental demand, time demand, performance, effort, and stress level	Feedback on the Idea generation and evolution parts using two discrete rates from 0 to 100 with 5-point steps
Excluded samples	Lack of concentration, participants requests, low data or biosignals quality, errors while recording the data	
The characteristics of the experiments	Experiments were loosely controlled in both datasets. Participants only received the steps of design and creativity. Participants were given free time with no interruption and no interference.	
Data preprocessing	Popular EEG preprocessing pipelines to remove probable EEG artifacts	

In both datasets, we set up loosely controlled experiments, including self-paced and open-ended tasks. Both datasets were recorded using a novel approach known as loosely controlled experiments, where only the design steps are controlled, allowing for self-paced, open-ended creativity. This method, more aligned with the nature of design tasks, yields more reliable and realistic results than traditional, strictly controlled experiments. Loosely controlled experiments balance scientific rigor with the natural flow of creativity, accommodating different problem-solving approaches and time requirements for design creativity tasks like idea generation and evolution. These experiments, occupying a middle ground between uncontrolled and strictly controlled, are tailored to specific research needs, ensuring scientific relevance. Loosely controlled experiments, in contrast to traditional approaches, align better with established creativity models, offering a less restrictive environment that fosters a deeper understanding of design interactions. These experiments also address the complexity of design tasks, providing hints to assist participants while allowing freedom in task execution. The reliability of loosely controlled experiments has been confirmed through comparisons with established research and various EEG analysis methods, including microstate analysis. This approach is effective for ecologically valid neurocognitive studies, extending EEG applications in design studies beyond basic stimulus-response relationships. While offering valuable insights, these experiments might pose challenges in EEG data processing due to the complexity of stimuli and responses. EEG microstate analysis has emerged as a solution, enabling precise segmentation of EEG data to match specific cognitive tasks, and enhancing data interpretation in these complex experimental settings. Loosely controlled experiments, typically longer than traditional ones, avoid extraneous controls, leading to more diverse design outcomes [1,2].

Both datasets were approved by the Concordia Human Research Ethics Committee. All steps of the experiments were performed according to the relevant regulations and guidelines. In both datasets, we monitored the whole experiment and stopped it at the participants' request or if there was an error during the procedure. We also used four cameras and recorded videos containing the experiment environment, the desk, and the computer that the participants used, their body movements as well as their faces. We used these videos to segment the EEG signals into their corresponding tasks. For the participants’ privacy, only preprocessed and segmented EEG signals are provided in the datasets and presented in this paper. It should be noted that we have also mentioned and cited the raw EEG recordings of the two datasets [10,11] as well. The two experiments were conducted at different times and were not consecutive. Different participants took part in the two experiments.

Both datasets were recorded using a 64-channel BrainVision (Brain Vision Solutions, Montreal, Canada) actiCHamp according to the 10-10 international system at a sampling frequency of 500 Hz. The names of the recorded channels are: Fp1, Fp2, Fpz, AF3, AF4, AF7, AF8, AFz, F1, F2, F3, F4, F5, F6, F7, F8, Fz, FT7, FT8, FC1, FC2, FC3, FC4, FC5, FC6, FCz, T7, T8, C1, C2, C3, C4, C5, C6, Cz, TP7, TP8, CP1, CP2, CP3, CP4, CP5, CP6, CPz, P1, P2, P3, P4, P5, P6, P7, P8, P9, P10, Pz, PO3, PO4, PO7, PO8, POz, O1, O2, Oz, Iz.. The two datasets are hosted on two separate Mendeley data repositories to let researchers employ either or both datasets in their future studies. Data A is stored on the design dataset (DOI:10.17632/h4rf6wzjcr.1) [12] and Data B is hosted on the creativity dataset (DOI:10.17632/24yp3xp58b.1) [13]. It should be noted that the raw EEGs corresponding to the two datasets A and B are also provided on Mendeley Data. The raw EEGs of the design dataset can be found at (DOI:10.17632/kpg948 × 794.1) [10] and raw EEGs of the creativity dataset can be found at (DOI:10.17632/82pgy2k6sk.1) [11].

To better understand the abbreviations used in the present paper, we decided to dedicate a table explaining the abbreviations. Table 2 defines and lists the abbreviations used in this paper, the corresponding full words, and their meanings.

**Table 2:** The abbreviations used in the present paper, their corresponding words, and explanations.

#	Abbreviation	Full word	Explanation
1	TTCT	Torrance Test of Creative Thinking	A creativity test
2	NASA-TLX	NASA Task Load Index	A tool to rate cognitive load
3	EEG	electroencephalography	The signals representing brain electrical activity
4	BDC	a birthday cake	A design problem which was used in Dataset A
5	REB	a recycle bin	A design problem which was used in Dataset A
6	TOB	a toothbrush	A design problem which was used in Dataset A
7	WHC	a wheelchair	A design problem which was used in Dataset A
8	WOS	a workspace	A design problem which was used in Dataset A
9	DRF	a drinking fountain	A design problem which was used in Dataset A
10	DP	design problem	An index representing the number of the design problem
11	PN	participants’ number	An Index representing participants’ number
12	TN	trial number	An Index representing the number of trials in the dataset B
13	PU	problem understanding	The cognitive task of understanding the design problem in dataset A
14	IG	idea generation	The cognitive task of generating a solution in dataset A
15	RIG	rating "idea genaration"	The cognitive task of rating the idea generation process in dataset A
16	IE	idea evaluation	The cognitive task of evaluating the solution in dataset A
17	RIE	rating "idea evaluation"	The cognitive task of rating the idea evaluation process in dataset A
18	RST1	rest time 1	The first 3 min rest at the beginning of the experiments
19	RST2	rest time 2	The second 3 min rest at the end of the experiments
20	IDG	idea generation	The cognitive task of generating ideas for creativity tasks in dataset B
21	IDE	idea evolution	The cognitive task of modifying ideas for creativity tasks in dataset B
22	IDR	idea evaluation or rating	The cognitive task of rating ideas for creativity tasks in dataset B
23	MARA	multiple artifact rejection algorithms	An EEG artifact removal method
24	HAPPE	Harvard Automated Processing Pipeline for Electroencephalography	An EEG artifact removal pipeline
25	PREP	standardized early-stage EEG processing pipeline	An EEG artifact removal pipeline
26	ICA	independent component analysis	A method of source separation in signal processing

Table 3 presents more details about the structure of the two datasets and how the EEG signals are stored and named. Dataset A contains a folder in which there are 27 files (associated with 27 participants) whose format is .mat. We named the files according to the participants’ numbers. Each participant was given six design problems (trials), each of which contained five design tasks. In Table 3, PN and DP stand for participants’ numbers and design problems, respectively. The design problems (trials) in this dataset are: designing 1) a birthday cake (BDC); 2) a recycle bin (REB); 3) a toothbrush (TOB); 4) a wheelchair (WHC); 5) a workspace (WOS); and 6) a drinking fountain (DRF). We used these abbreviations to briefly name the files in the data repository. Participants’ number (PN) ranges from 1 to 27, where DP ranges from 1 to 6, representing the number of the assigned design problems. In each of these design problems, participants performed five design tasks, including problem understanding (PU), idea generation (IG), rating the generated idea (RIG), idea evaluation (IE), and rating idea evaluation (RIE). Each experiment also included two 3 min eye-closed rest periods at the beginning and end of each experiment, which are represented by RST1 and RST2, respectively. So, in dataset A (design dataset), each (.mat) file contains 32 variables (for each participant) which are the EEG recordings of the six design problems (BDC, REB, TOB, WHC, WOS, and DRF), each of which included five design tasks (PU, IG, RIG, IE, and RIE) and two rest periods (RST1 and RST2) at the beginning and the end of the whole experiment.

**Table 3:** folder and file details in datasets A and B.

Dataset	Dataset Title	Root Folder	Files’ name	Data Structure (Variables’ names representing EEG signals stored in the .mat files)
Design Dataset (A)	Conceptual Design Exploration: EEG Dataset in Open-ended Loosely Controlled Design Experiments	Design_EEG_Dataset	Data_Design_Sub_PN (.mat)	Design_[PN]_[DP]_PU Design_[PN]_[DP]_IG Design_[PN]_[DP]_RIG Design_[PN]_[DP]_IE Design_[PN]_[DP]_RIE Design_[PN]_[DP]_RST1 Design_[PN]_[DP]_RST2
Creativity Dataset (B)	Design Creativity: EEG Dataset in Loosely Controlled Modified TTCT-F Creativity Experiments	Creativity_EEG_Dataset	Data_Creativity_Sub_PN (.mat)	Creativity_[PN]_[TN]_IDG Creativity_[PN]_[TN]_IDE Creativity_[PN]_[TN]_IDR Creativity_[PN]_[TN]_RST1 Creativity_[PN]_[TN]_RST2

In Table 3, the creativity dataset is also represented. Dataset B, which represents EEG signals recorded during creativity experiments, contains one folder (“Creativity_EEG_Dataset”) in which there exist 28 (.mat) files. These files are associated with and named according to the 28 participants in the creativity experiments. There were three trials associated with three different stimuli in each experiment. In each trial (run), participants were asked to perform three creativity tasks, including 1) idea generation (IDG): intuitively completing a sketch based on their initial perception of a given image, 2) idea evolution (IDE): creating a drawing that significantly diverged from the previous sketch, and 3) idea evaluation or rating (IDR): assessing the two previous tasks on how difficult it was to think and draw. Like the design dataset, there are two 3 min rest periods at the beginning and end of each creativity experiment, which are named RST1 and RST2, respectively.

In Table 3, we used a procedure to name the files and variables in the two datasets. To name the variables in the design dataset we employed a pattern as [“Design”]_[participant number]_[design problem]_[task]. In the creativity dataset, we used [“Creativity”]_[participant number]_[trial number]_[creativity task] to name the variables. PN, DP, and TN stand for the participant's number, design problem, and trial number, respectively. As mentioned before, in the design dataset, for each participant (PN), there are 6 design problems (DPs), each of which contains five design tasks (PU, IG, RIG, IE, and RIE) and two rest periods (RST1 and RST2). In the creativity dataset, for each participant (PN), there are three trials (TNs), each of which contains three creativity tasks (IDG, IDE, and IDR) and two rest periods (RST1, RST2) at the beginning and end of each experiment. We used these indices to represent participants, design problems, and trials, facilitate coding, and also to make the datasets easier to use for future studies. All the EEG signals corresponding to design or creativity problems can be loaded in one folder in an organized way and without duplicate names.

In both design and creativity experiments, participants were initially given the following instructions at the start of the experiments:

1. You are allowed to use an electronic tablet to express/sketch your design ideas and to make notes.
2. You are free to go back and forth to check your previous sketches/notes at any time.
3. You will not be interrupted during the design task.
4. Once the experiment starts, you are not supposed to ask any questions related to the design. However, you can ask for assistance regarding the tools you are using for design.
5. You would not be able to use the Internet during the design process.
6. Work as you would normally do.

In both experiments, participants engaged in a series of tasks using a graphical user interface (GUI), which displayed instructions for each step of the design process, including task performance and subsequent ratings. Participants could proceed to the next step by clicking “Next” on the screen.

In the design experiment, the specific instructions provided to participants are outlined as follows:

1. Problem Understanding: Participants were presented with six design problems, as previously detailed. The problems were displayed on the GUI, and participants were instructed to click the “Next” button once they had clearly understood each problem. The instruction was, “A problem is presented to you. After you read the problem and understand what needs to be done, click Next to move to the next stage.”
2. Idea Generation: For the second step, participants were given access to a blank drawing board to visually represent their generated ideas. The instruction was, “A note-taking application will pop up. Use the tablet pen to write your solution. After you finish, save, and close the file.” After closing the drawing application, the GUI displayed the message “Click Next to continue.”
3. Rating Idea Generation: Participants were then asked to rate their experience of the idea generation process. The NASA-TLX was presented to participants for them to rate their experience of the previous step. Participants were asked to rate their mental demand (How mentally demanding was the task? How much thinking does the task require?), time demand (How much time pressure did you feel? Do you feel that you need more time to complete the design?), performance (How satisfied are you with your performance?), effort (How hard did you have to work?), and stress level (How uncertain, confused, uneasy did you feel?). The rating was conducted using a 0 to 100 point scale with a 5-point interval, where 0 indicated “low” and 100 represented “high”. The instruction stated, “For each factor, choose the point that best indicates your experience of the SOLUTION GENERATION process. Please click on “Next” when you finish the rating.”
4. Idea Evaluation: This step involved presenting participants with two potential design solutions, labeled “Solution 1” and “Solution 2,” displayed on the left and right sides of the GUI, respectively. The instruction for this step was, “Which solution is better (or which solution do you prefer)? Why?”. Below the two design options, a space was provided for participants to indicate their preferred solution and explain their choice.
5. Rating Idea Evaluation: Finally, participants were asked to rate their experience of the idea evaluation process using the same set of questions (NASA-TLX) and rating scale as in step 3. The instruction was, “For each factor, choose the point that best indicates your experience of the “SOLUTION EVALUATION” process.” The GUI displayed the same rating system as used in the idea generation rating step.

In the creativity experiments, similar to the design dataset, a graphical user interface (GUI) was utilized to provide instructions to participants and facilitate the rating of the process. The specific instructions given to participants in the design experiment were as follows:

1. Idea Generation: Participants were tasked with generating creative sketches based on an initial sketch provided to them. The exact instruction given was, “What do you see from the figure below? Draw it and give it a title.”
2. Idea Evolution: In this phase, participants were instructed to modify their initial sketch. The goal was to encourage creativity and uniqueness in their solutions. The instruction for this step was, “Complete the picture (creatively) so that your solution would not look similar to others’ and the previous drawing. Give it a title.”
3. Idea Evaluation: This step involved participants rating the process based on two questions: “How difficult is it for you to think of the image?” and “How difficult is it for you to draw the image?”. The rating options provided were “Easy” and “Difficult,” each with four degrees of intensity: “extremely”, “very”, “quite”, and “slightly.” Additionally, participants had the option to select “Neutral” instead of “Easy” or “Difficult.” This rating was provided using a discrete scale ranging from “extremely easy” to “neutral” and to “extremely difficult”.

To better explain the cognitive tasks included in design and creativity tasks, we decided to give a short yet clear description of these tasks. This will help to better understand what the EEG data can reflect about the cognitive processes of each step such as problem understanding, idea generation, etc. in these experiments. In the realm of design research, whether it be in engineering, architecture, or product design, the designer leverages their specialized knowledge, encompassing aspects like dimensions, style suitability, and material selection, to ideate and assess solutions aimed at achieving specific objectives. This process unfolds within various constraints, including budget limitations, client requirements, etc. The design process is characterized by a sophisticated interplay between two types of thinking: knowledge-driven or goal-driven (top-down) thinking and environmentally-driven or data-driven (bottom-up) thinking. Top-down thinking is guided by the designer's expertise and the objectives of the project, while bottom-up thinking is influenced by external data and environmental factors. The ability of a designer to effectively oscillate between these two modes of thinking, determining when to apply each, plays a crucial role in their success. This balance is key to solving design problems in a manner that is not only creative but also efficient. Psychological research into the creative process sheds light on how designers balance their existing knowledge, past experiences, and comprehension of design goals with the necessity to develop innovative and unique solutions. Creative ideation, the generation of various original ideas for open-ended problems, is understood as a cognitive activity that involves retrieving existing knowledge and combining different aspects of this knowledge into new ideas. This process encompasses two primary cognitive functions: divergent and convergent thinking. Divergent thinking, which is linked to higher-level cognitive processes like memory retrieval, semantic associations, and mental imagery, is about generating a range of solutions. Conversely, convergent thinking focuses on finding a specific solution within a limited set of acceptable answers, relying on routine mental operations, and demanding more concentration and persistence. Divergent thinking tasks, often referred to as creative ideation tasks, imply exploring multiple potential solutions of varying quality, as opposed to convergent thinking tasks that aim for a single correct answer. During divergent thinking, internal attention increases, blocking irrelevant external stimuli and focusing on internal mental processes. This type of thinking relies more on internal processing, as common solutions are more easily retrieved due to their stronger associative links. Creative idea generation involves inhibiting irrelevant stimuli and enhancing internal focus, a phenomenon supported by creativity models and EEG alpha band analysis in creativity studies. The observed increase in alpha power during creative ideation may indicate a more internally focused attention, characterized by reduced external stimulation and the inhibition of irrelevant cognitive processes, reflecting a top-down activity. It might also involve specific memory processes like efficiently combining unrelated semantic information. Therefore, internal attention and task inhibition are key cognitive states in idea generation. In convergent thinking, the objective is to identify the most direct association between a stimulus and its closest semantic representation. This process involves shifting attention to external stimuli and maintaining focus on the tangible aspects of the object, such as its conventional use. The first step is “problem understanding” where participants need to read the design problem, digest the requirements, focus on the ambiguities of the design task, percept the constraints and probable conflicts existing in the design problem. For example, as mentioned above, in designing a water fountain, the problem contains a conflict as “(a) filling up a water bottle is not easy; (b) people too short cannot use the fountain and people too tall have to bend over too much”. The cognitive tasks during problem understanding mainly include comprehension, analysis, and critical thinking. During idea generation, participants compared similar design problems with their prior personal knowledge using memory retrieval, using their creativity, synthesizing their design, and visualizing it. Problem definition, memory retrieval, divergent thinking, imagination, idea assessment, new comprehension of the design problem, convergent thinking, synthesis, and visualization are the main cognitive tasks involved in idea generation. During idea evaluation, memory retrieval, critical analysis of solutions, and self-assessment are the main cognitive tasks one should perform. In creativity experiments, idea evolution involves several cognitive tasks such as divergent thinking, analysis, memory retrieval, imagination and visualization, critical thinking and evaluation, metacognition, and decision-making [1,2,4,6,8,9].

We decided to offer a detailed comparison of the two datasets, examining their differences and similarities. We present a direct, side-by-side analysis of their key characteristics, aiming to clearly distinguish and emphasize their unique and common attributes.

The main difference between the two datasets is their protocols. While the design dataset includes a novel approach to define design tasks, the creativity dataset is more based on a well-known and popular protocol called the verbal version of TTCT. In dataset A, participants draw their designs and rate them. All steps such as the process of idea generation and the process of evaluating the two given solutions were also rated by participants. These rating processes were handled using NASA-TLX which is a popular rating tool for cognitive abilities. The design problems were quite novel and contained the criteria that possible solutions should have as design requirements. The creativity dataset was less controlled compared to the design dataset in the sense that participants were instructed with a short instruction according to which they generated their creative ideas. The whole protocol was standardized since the figural version of TTCT was used compared to the design dataset where novel design problems were employed. On the other hand, the evaluation processes of the design dataset were more structured as the standard NASA-TLX was used to fully rate the cognitive workload considering different aspects. In contrast, the evaluation process of the creativity dataset was performed through a novel tool including two questions regarding the cognitive aspects of creativity.

The design dataset included five design tasks which contained several cognitive tasks such as understanding, creating, remembering, testing, applying, analyzing, and evaluating. On the other hand, the creativity experiments included three steps and relied more on two cognitive states called divergent thinking (idea generation) and convergent thinking (idea evolution). This is another major difference in terms of cognitive states involved in these two datasets.

Both datasets included almost the same number of participants and followed a quite similar approach for EEG signal pre-processing. In both datasets, popular EEG artifact removal pipelines such as HAPPE and PREP were utilized to ensure the quality of the EEG pre-processing phase. Generally speaking, in most cases, EEG signals in the design dataset were a bit longer than the ones in the creativity dataset. One reason could be the number of steps and the nature of design and creativity experiments. Considering the two pre-processed EEG datasets, both datasets have the potential to be used in neuroscientific and/or machine learning-based projects in the future.

In this section, we tried to provide some information about the two datasets’ description, the experiments and their corresponding cognitive tasks, the datasets’ format, and how they are stored in the data repositories. It is worth mentioning that all the variables (EEG signals) are presented in 2D matrixes whose rows represent the number of channels, which is 63 in these two datasets, as during preprocessing, all the 64 EEG channels were referenced to the Cz channel which was finally removed as the reference channel. This resulted in 63 channels stored in the two datasets, where the rows represent the number of channels, and the columns represent the length of the EEG signals, which are variable according to the self-paced and open-ended design and creativity tasks.

## 3 Experimental Design, Materials and Methods

### 3.1 Dataset A

#### 3.1.1 Participants

In this dataset, 42 individuals who were graduate students at Gina Cody Engineering School and Computer Science, participated in the experiments. All participants had normal or corrected-to-normal vision, reported mental health, and had no history of medical or psychiatric disorders or treatment. None of the participants reported sleep deprivation during the past few days or a history of drug abuse. In addition, all participants were asked not to consume alcohol or caffeinated drinks before participating in our experiments. Three, eleven, and one participant were excluded from the experiment due to not finishing all the experiments, some technical errors, and recorded biosignals with poor quality, respectively. EEGs of poor quality were identified by visually inspecting both time and frequency domains. Participants were excluded if severe noise was detected in either domain. Additionally, electrode-skin impedance was monitored, excluding participants with impedance exceeding 10 KΩ. Finally, EEG signals from 27 participants (8 women) were stored in dataset A. These participants were aged from 24 to 39.

#### 3.1.2 Experiment setup

The experimental setup included a comfortable chair, a computer desk, a computer, a monitor, and a 15-inch Wacom drawing tablet (Wacom Cintiq 15X LCD Tablet, Model: PL-550, Taiwan), which participants used to draw their designs. The room was well-lit and quiet. The experiments’ guidelines and tasks were shown to the participants on the computer screen. The research technicians used another computer to monitor the experiment, record videos, and record EEG signals. The EEG recording system was the 64-channel BrainVision (Brain Vision Solutions, Montreal, Canada). The EEG recording device was connected to that computer through a USB dongle. A divider was used to separate the participants from the research technicians. Four cameras were used to record videos of the experiments, the participants’ activities, drawings, and behavior. These videos were later used to preprocess and segment EEG signals. It is worth mentioning that design tasks, the environment, and the recording systems were tested by some volunteers who did not participate in the main experiments to make sure about the quality of our experiments. An experiment logbook was used to record all the details of the experiments.

#### 3.1.3 Experiment protocol and procedure

**Fig. 1:**
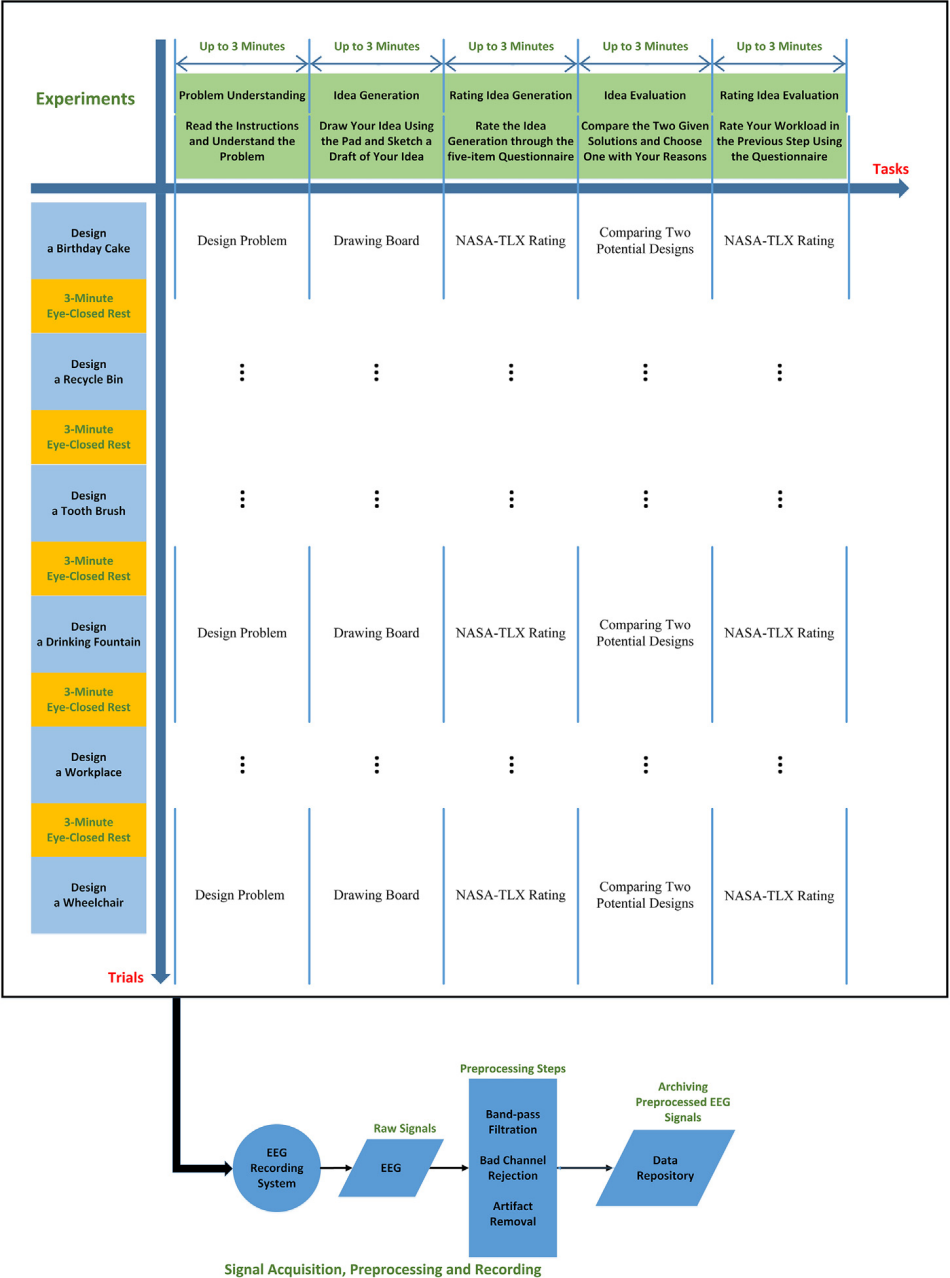
The block diagram of the experiments in the design dataset.

Fig. 1 represents the procedure of the design dataset. Twenty-seven participants took part in this experiment. Participants were asked to read and sign the consent form after being informed about the experiments and procedures. Then, the guidelines for the experiment were explained and provided for the participants. Their questions were answered by the research technicians, if any. Only once the participants agreed and signed the consent did the experiment start. The monetary compensation for the best design was CAD $100. Participants were told the experiment would be stopped immediately at their request at any stage if they felt uncomfortable.

Participants were asked to sit comfortably on the chair in an upright position facing the computer screen, and the EEG cap was placed according to the 10-10 international standard system over the participants’ heads. Their heads’ diameter was measured by a flexible tape measure to determine which EEG cap size should be used. After placing the EEG cap, the Cz channel was checked to be placed on the vertex of the head. We used a syringe with conductive gel to reduce the impedance of the contact between the scalp and the electrodes. The electrode-skin impedance was checked to be below 10 KΩ by the EEG recording device, and the quality of EEG signals was frequently checked through visual inspection. The EEG recording system was checked to be working appropriately. The electrodes were also manually checked for appropriate contact to make sure about the high quality of the EEG signals. EEG signals were recorded during the whole experiment and later preprocessed and segmented. Each experiment included six design problems, each of which consisted of five open-ended, self-paced design tasks, including problem understanding, idea generation, rating idea generation, idea evaluation, and rating idea evaluation. Three-minute closed-eye rests were considered at the beginning and end of each experiment.

The design problems were as below:

1. “Design a birthday cake: Make a birthday cake for a 5-year-old kid. How should it look like?”
2. “Design a recycle bin: Sometimes, we do not know which items should be recycled. Create a recycle bin that helps people recycle correctly.”
3. “Design a toothbrush: Create a toothbrush that incorporates toothpaste (incorporate = include, combine).”
4. “Design a wheelchair: In Montreal, people on a wheelchair cannot use the metro safely because most of the metros have only stairs or escalators. An elevator is not an option because it is too costly to build one. You are asked to create the most efficient solution to solve this problem.”
5. “Design a workspace: Employees in an IT company are sitting too much. The company wants their employees to stay healthy and work efficiently at the same time. You are asked to create a workspace that can help the employee to work and exercise at the same time.”
6. “Design a drinking fountain: Two problems with standard drinking fountain: (a) filling up a water bottle is not easy; (b) people too short cannot use the fountain and people too tall have to bend over too much. Create a new drinking fountain that solves these problems” [1,2].

Each design problem contains five tasks, including problem understanding, where participants are instructed to read the given design problems; idea generation, where volunteers are asked to draw their primitive solution that satisfies all the problem's requirements; idea evaluation, where they are asked to analyze and rate the performance of two given solutions. We put the NASA Task Load Index [14] at the end of the idea generation and evaluation steps and asked participants to report their mental demands, time demands, performance, effort, and stress levels. The NASA Task Load Index evaluates workload through six 7-point scales: performance, effort, stress level, physical demand, mental demand, and time demand. In our study, physical demand was not considered as the whole experiment included cognitive and mental tasks.

Dividing design problems was performed by considering five open-ended tasks in each design problem to decrease the complexity of the whole design process. It was the main and only structure we placed in the whole experiment to help participants with the design problems. Due to the advantages of loosely controlled design experiments [1,2] we decided to avoid almost all additional constraints and allow participants to spend as much time as they wanted without interruption or interference to let them perform the creativity tasks as naturally as they do in real-world situations. By giving sufficient time and freedom, we are more likely to design experiments with more natural characteristics, as real experiments do.

#### 3.1.4 EEG signal preprocessing and segmentation

Recorded EEG signals were stored in a computer rather than the one used for the experiments. EEG signals were associated with the participants’ numbers. Recorded EEG signals were checked and preprocessed using EEGLAB [15]. We used the HAPPE pipeline [16] to preprocess the EEG signals. Our EEG preprocessing had four main steps, including 1) bandpass FIR zero phase windowed-sinc Hamming filter between 1 and 40 Hz; 2) detecting and isolating bad global EEG channels; 3) removing common biological artifacts such as eye blink, muscle-generated artifacts, eye movement, etc.; and 4) detecting and interpolating bad local channels through segmenting EEGs into 2-s epochs. This segmentation was used to detect bad local channels and EEG segments [16]. Recognizing bad global channels was conducted according to the following procedure: An EEG channel was labeled as a bad global channel if it was flat for more than 5 s, or if the correlation coefficient between this channel and neighboring channels was smaller than 0.8, or if the subtraction of the channel's amplitude from the signal mean was greater than three standard deviations. Biological artifacts were recognized and eliminated using multiple artifact rejection algorithms (MARA) [16] in EEGLAB when independent components extracted from EEG had more than a 40% chance of being classified as artifacts. EEG channels were segmented into 2 s windows, and FASTER [17] criteria were employed to detect bad local EEG channels. Variance, amplitude range, gradient, median, and deviation from the signal mean were considered as four criteria to recognize bad local EEG channels. One or more Z-scores higher than three standard deviations from the mean showed that the corresponding EEG segment should be assumed to be a bad local EEG channel, which later would be interpolated using spherical splines. To identify bad EEG segments, the following criteria were employed: a channel was considered a bad channel if its amplitude exceeded ±100 μV, or if the single electrode probability across all segments of the electrode group probability within segments deviated by more than three standard deviations from the mean. Subsequently, we interpolated the isolated bad global channels through spherical splines. The preprocessed EEG signals were then re-referenced to the average reference and down-sampled to 250 Hz. Finally, the EEG signals were segmented using video recordings of the experiments. The preprocessed and segmented EEGs were stored in the dataset.

### 3.2 Dataset B

#### 3.2.1 Participants

Participants were 29 graduate students from the Concordia Institute for Information Systems Engineering, Concordia University. All participants had normal or corrected vision, reported good mental health, and had no history of medical or psychiatric issues or treatment. None of them had experienced recent sleep deprivation or drug abuse. All participants were in normal health and mental condition and were informed about the experiment and its steps. We also asked them not to drink alcohol or caffeinated beverages before participating in our experiments. One of the participants was removed from the dataset due to poor quality and noisy EEGs. Poor EEGs were detected through visual inspection of both time and frequency domains. The presence of severe noise in one or both of these domains was the exclusion criterion for participants. In addition, the electrode-skin impedance was also checked and we would exclude a participant if the impedance was greater than 10 KΩ. Finally, EEG signals from 28 participants were recorded and stored in this dataset. Twenty-eight right-handed participants (4 women), aged from 22 to 35, took part in the experiments and received an oral explanation of the experiment, including details about the tasks, the EEG recording process, and the experiment's procedure.

#### 3.2.2 Experiment set-up

Similar to dataset A, in dataset B, a comfortable chair, a computer desk, a computer, a monitor, and a 15-inch Wacom drawing tablet (Wacom, Taiwan) were used in the creativity experiment as well. The tablet's name and model were Wacom Cintiq 15X LCD Tablet, Model: PL-550, respectively. We used the computer screen to send the commands or instructions to the participants. Their drawings were captured by recording the screen of the Wacom drawing tablet. Four cameras were used to monitor the participants, their behavior, drawings, and the whole experiment. Later, this video data was employed to segment EEG signals according to their corresponding creativity tasks. Similar to the design dataset, technicians used another computer to observe the experiment and record EEG signals. The room was quiet and well-lit. A divider was employed to create a physical separation between the participants and the technicians and monitoring systems. Volunteers who were not a part of the main study tested the environment and recording systems to ensure the quality of our experiments. We used an experiment logbook to document all experiment-related details. We used the same EEG recording system as the design dataset. EEG signals were captured by a 64-channel BrainVision (Brain Vision Solutions, Montreal, Canada) according to the 10-10 international system with a sampling frequency of 500 Hz. The resolution of the EEG recording systems was 24 bits. All channels were referenced to the Cz channel being later removed resulting in 63 channels of EEG stored in the creativity dataset.

#### 3.2.3 Protocol and procedure

**Fig. 2:**
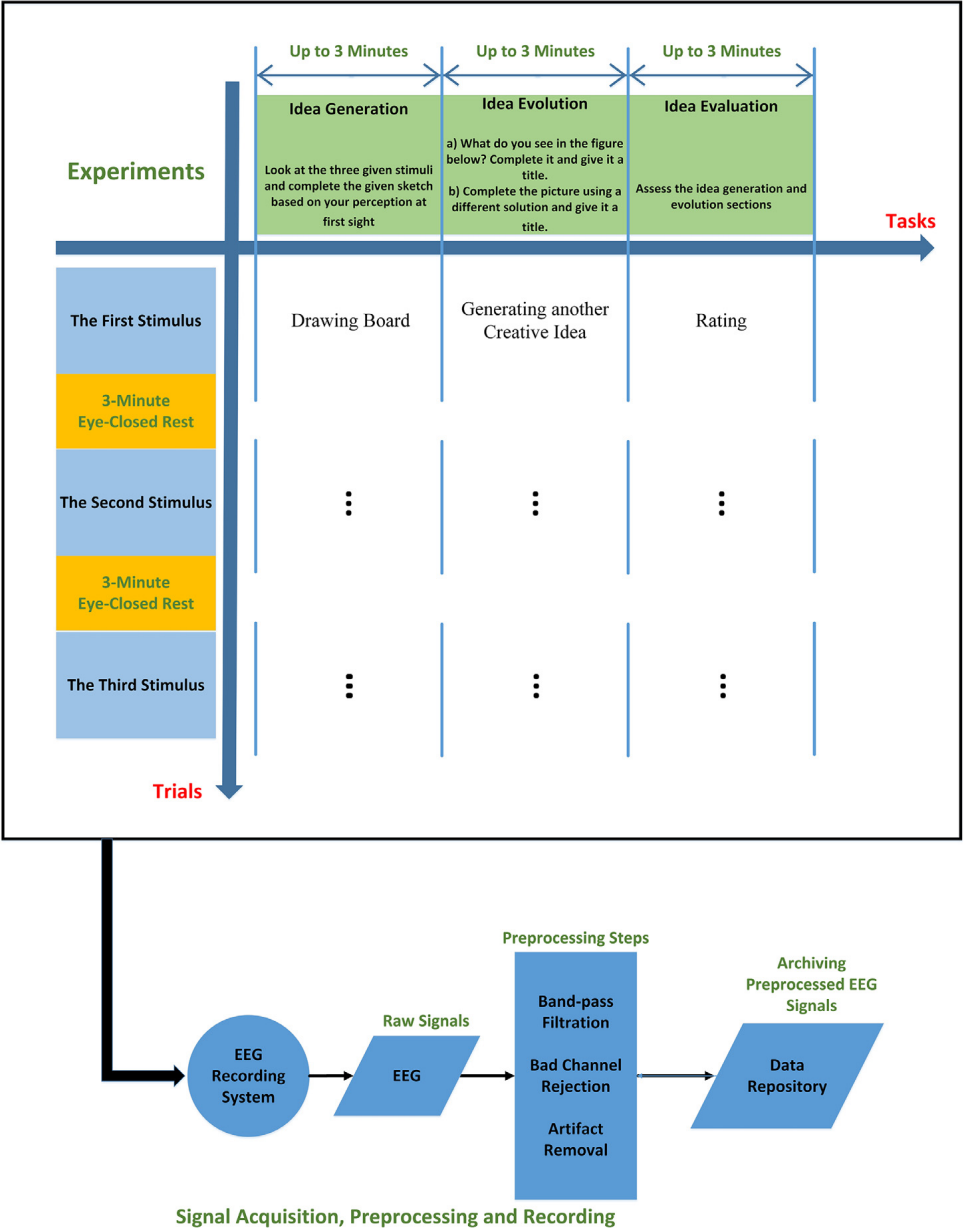
The block diagram of the experiments in the creativity dataset.

Fig. 2 illustrates the procedure of the creativity experiments. Participants read and signed the informed written consent forms and then took part in the experiments. As mentioned above, the initial number of participants was 29, and later we removed one participant due to the poor quality of the EEG signals. So, we finally recorded and stored EEG signals from 28 participants. Potential participants were contacted and asked to take part in the experiment. Technicians explained the procedure of the experiment and answered participants’ questions. Participants were informed that they could request the immediate cessation of the experiment at any point if they felt uncomfortable. Participants received a gift card valued at CAD$15 after completing the experiment as a gesture of gratitude for their participation. Once we obtained the signed consent forms, we started to prepare the participants for the experiment. Participants were instructed to sit comfortably in an upright position, facing the computer screen. Following this, the EEG cap was positioned in accordance with the 10-10 international standard system, tailored to the participants' head size (small, medium, or large, determined by head diameter). After cap placement, the Cz channel was verified to align with the vertex of the head. To ensure optimal contact between the scalp and the electrodes, we applied a conductive gel using a syringe. Electrode impedance was regularly monitored to ensure it remained below 10 KΩ, as verified through the EEG recording device. Additionally, the quality of EEG signals was visually inspected. System functionality checks were performed to confirm that the EEG recording system was operating correctly. Both the device and electrodes were manually inspected to ensure proper contact, thereby ensuring the high quality of the EEG signals. EEG signals were recorded throughout the experiments and subsequently underwent preprocessing and segmentation.

The experiment started when we made sure that the recording systems, including the EEG recording device and cameras, were working appropriately. Our proposed procedure for the creativity experiment was derived from the modified figural Torrance Test of Creative Thinking (TTCT-F) test and a study on sketch evolution [3,18]. Each creativity experiment included three creativity stimuli (or problems) where the participants were given three incomplete sketches. These sketches are assumed to be stimuli for creativity. These initial and incomplete sketches can be seen in Fig. 2. In each run, participants were given a creativity problem. Each run consisted of a sequence of idea generation, idea evolution, and evaluation [3], repeated three times. We used simple windows (Graphical User Interfaces) to send the commands and guidelines to the participants. In each run, participants were asked to perform three creativity tasks, including idea generation, idea evolution, and idea evaluation (rating). Therefore, the experiment consisted of three runs and three creativity tasks within each run. In the idea generation section, participants were instructed to intuitively complete a sketch based on their initial perception of the image. Subsequently, during the idea evolution phase, volunteers were tasked with creating a drawing that significantly diverged from the previous sketch, reflecting an evolved idea. In the third section, participants were instructed to assess the idea generation and evolution sections based on how difficult it was to think and draw. In the idea evaluation phase, we asked two questions, including: “How difficult is it for you to think of the image?” and “How difficult is it for you to draw the image?”. Participants used a discrete rating which ranged from 0 to 100 to answer these two questions. It is worth mentioning that in this experiment, like design experiments, we also aimed to provide flexibility and freedom for participants to have more natural and reliable experiments, and all three creativity tasks were loosely controlled (self-paced and open-ended up to three minutes). Each section lasted up to 3 min, and three-minute rests were considered at the beginning and end of each experiment.

In the creativity experiments, we took an approach quite similar to the design dataset. Although we used the same experiment setup in both design and creativity datasets, there are major differences in the given problems and the assigned cognitive tasks. It should be noted that the participants, assigned problems, and procedures in the creativity experiments were different from the ones in the design dataset.

#### 3.2.4 EEG signal preprocessing

Similar to dataset A, EEG signals were preprocessed using the Harvard Automated Processing Pipeline for Electroencephalography (HAPPE) [16]. First, a bandpass filter with a bandwidth of 1 to 50 Hz was employed. Global bad EEG channels were recognized and isolated through the standardized early-stage EEG processing pipeline (PREP) [19]. Third, artifacts, including biological artifacts and high-amplitude ones, were eliminated from EEG signals using independent components analysis (ICA) and wavelet transformation through the following processHAPPE incorporates a wavelet-enhanced ICA method as a preliminary step to address EEG artifacts while maintaining the full length of the EEG recording. This is followed by a traditional ICA process to eliminate artifact components. The W-ICA step is designed to remove various types of artifacts, including those generated by eye movements and muscles, high-amplitude artifacts such as blinks, and signal discontinuities. The process involves initially decomposing the EEG signal into components through ICA. Subsequently, each EEG component undergoes a wavelet transform and is later thresholded to eliminate artifacts. The processed signals are then reconverted into the EEG channel format. In this phase, while artifacts within each ICA component are targeted and removed, no components are entirely discarded. The threshold is determined automatically using the signal's components, their variance, and length, as follows:

(1) $$W-ICA\;Threshold\;=\;\frac{median\;\left(abs\left(C\right)\right)}{0.6745}\;*\;\sqrt{2\;\log\left(L\right)}\;$$

Where C is the set of detailed coefficients provided by the wavelet transform, and L is the length of the ICA components. The wavelet-enhanced ICA is followed first by a traditional ICA, then by the Multiple Artifact Rejection Algorithm (MARA), and finally by re-referencing to average reference [16].

Then, local bad channels within each 2 s segmented epoch of the EEG data were identified and isolated using FASTER [17]. EEG data epochs were retained for analysis when the ratio between the number of bad channels and all channels was less than 0.25. Global and local bad channels were interpolated from nearby channels [20]. On average, the mean number of removed bad epochs was 0.964 (SE = 0.358) for rest, 0.214 (SE = 0.065) for idea generation, 0.345 (SE = 0.085) for idea evolution, and 0 (SE = 0) for evaluation. The average number of interpolated globally bad channels was 5.357 (SE = 0.593). At the end, the EEG data were re-referenced to an average reference. Ultimately, the EEG signals were segmented by aligning them with video recordings of the experiments. Then, the preprocessed and segmented EEG data were stored in the creativity dataset. It is worth noting that the sampling frequency of the EEGs in the creativity dataset is 500 Hz, whereas the final sampling rate of the EEG signals in the design dataset was 250 Hz.

### 3.3 Data and code availability

Since pre-processing and segmentation of the EEG signals require synchronized EEG and video processing, and also in videos the participants’ faces might appear, we decided to upload the preprocessed and segmented versions of the EEG signals for both datasets. These cleaned and segmented EEG signals are much simpler to use. We used popular EEG preprocessing pipelines and methods such as [16,17] to remove artifacts and probable noises. The EEGLAB toolbox, which was used for EEG preprocessing, can be found in [15]. The EEG signals of the design dataset (dataset A) can be found in Mendeley data (DOI:10.17632/h4rf6wzjcr.1) [12]. The preprocessed and segmented EEG signals for the creativity dataset are also stored in a different Mendeley data repository (DOI:10.17632/24yp3xp58b.1) [13]. Since clean and segmented EEGs are provided in both datasets, users can directly employ the datasets in their studies. It should be noted that the raw EEG recordings of both design and creativity datasets have also been uploaded to Mendeley Data. Raw EEG recordings associated with the design experiments can be found in [10] and raw EEG recordings associated with the creativity experiments can be found in [11].

### 3.4 Example usage of the EEG data

Two papers [1,2]*,* authored by our research team, have employed the datasets. The first paper [2] used the EEG signals in the design dataset to explore the brain dynamics in design activities. In this paper, the authors used EEG analysis to identify large-scale brain networks and understand their temporal dynamics during different design activities. The results showed that idea generation had the highest cognitive workload and the lowest cognitive control compared to other design tasks. EEG microstate analysis supported this, indicating greater freedom in brain network configurations during idea generation compared to other design activities. In the second study [1], the authors used microstate analysis to process EEG signals recorded during creativity tasks. The findings showed decreased alpha power during all thinking modes, with idea evolution requiring less general attention compared to the others. EEG microstate analysis revealed different brain network activations, with the default mode network more active during idea evolution and the cognitive control network more active during evaluation. In both papers, the results were consistent with most previous studies analyzing brain activities in design and creativity cognitive tasks. Although these two studies have deeply analyzed EEG signals in design and creativity tasks, there also exist some earlier studies, such as [3,4] which analyzed the two datasets and provided foundations for future studies.

## Limitations

Recording EEG signals in design and creativity experiments presents significant challenges. These experiments are characterized by their ill-structured, open-ended nature and extended duration. Prolonged EEG recording poses difficulties, including data loss and diminished recording quality. Lengthy EEG signals are susceptible to noise and various artifacts, particularly movement artifacts, given the physical nature of design activities. Consequently, recording and preprocessing EEG signals has become a complex task. Design and creativity experiments often span several minutes to hours, limiting participant willingness and resulting in a reduced pool of potential contributors. Additionally, the discomfort associated with wearing an EEG cap for extended periods can impact both data quality and participant concentration on tasks. These factors collectively contribute to the daunting nature of EEG recording in design and creativity experiments. For instance, in our design dataset, EEG recordings from three participants were excluded due to unfinished experiments, eleven were removed due to technical errors, and one was omitted due to poor EEG quality. These instances underscore the practical challenges and potential limitations inherent in EEG data collection in such experimental contexts.

## Ethics Statement

For both datasets, all the participants read and signed a written informed consent form. Both datasets were approved by the Concordia Human Research Ethics Committee. The ethical approval number is 10000041. All the experiments followed the ethical guidelines from the Declaration of Helsinki. All steps of the experiments were performed according to the relevant regulations and guidelines. All participants were instructed about the experiments, and informed consent was obtained from them.

## Declaration of Generative AI in Scientific Writing

During the preparation of this work, the authors used ChatGPT (OpenAI) to improve readability and language. After using this tool, the authors reviewed and edited the content as needed and take full responsibility for the content of the publication.

## CRediT authorship contribution statement

**Morteza Zangeneh Soroush:** Writing – original draft, Visualization, Data curation, Writing – review & editing, Software. **Mengting Zhao:** Visualization, Data curation, Writing – review & editing, Software, Validation. **Wenjun Jia:** Software, Formal analysis, Validation, Writing – review & editing. **Yong Zeng:** Conceptualization, Methodology, Supervision, Project administration, Funding acquisition, Writing – review & editing.

## Data Availability

Conceptual Design Exploration: EEG Dataset in Open-ended Loosely Controlled Design Experiments (Original data) (Mendeley Data) Design Creativity: EEG Dataset in Loosely Controlled Modified TTCT-F Creativity Experiments (Original data) (Mendeley Data) Design Creativity Raw EEG Recordings - Loosely Controlled Modified TTCT-F Creativity Experiments (Original data) (Mendeley Data) Conceptual Design Raw EEG Recordings - Open Ended Loosely Controlled Design Experiments (Original data) (Mendeley Data)

## Appendix Supplementary materials

Image, application 1
